# Supplementary material for: The impact of diabetes on visual acuity in Ethiopia, 2021
Source: PLoS One. 2021 Aug 13;16(8):e0256145. doi: 10.1371/journal.pone.0256145 (PMC8362981; doi:10.1371/journal.pone.0256145)
Supplement: S1 Table — (DOCX) [file pone.0256145.s001.docx]

S1 Table. The univariate analysis of visual impairment of diabetic patients.

| Outcome | Variables | Category | Coef. (β) | p-value | 95% CI |
| --- | --- | --- | --- | --- | --- |
| Blind | Sex | Female (R) |  |  |  |
|  |  | Male | 0.12 | 0.2376 | -0.37, 0.61 |
|  | Place of residence | Rural (R) |  |  |  |
|  |  | Urban | 0.07 | 0.9920 | -0.42, 0.55 |
|  | Age_cat | 17-39 (R) |  |  |  |
|  |  | 40-64 | 0.46 | 0.0001 | -0.51, 1.43 |
|  |  | >64 | 1.11 | 0.0001 | 0.15, 2.08 |
|  | Family history | No (R) |  |  |  |
|  |  | Yes | -0.70 | 0.5479 | -1.71, 0.31 |
|  | Type of diabetes | Type 1 (R) |  |  |  |
|  |  | Type 2 | 2.19 | 0.0000 | 1.12, 3.27 |
|  | Hypertension | No (R) |  |  |  |
|  |  | Yes | 0.67 | 0.0995 | -0.01, 1.34 |
|  | Duration of diabetes | 10 years and above (R) |  |  |  |
|  |  | Less than 10 years | -0.84 | 0.0000 | -1.37, -0.31 |
|  | Retinopathy | Non-proliferative (R) |  |  |  |
|  |  | Proliferative | 6.85 | 0.0000 | 0.81, 8.88 |
|  | Maculopathy | No (R) |  |  |  |
|  |  | Yes | 5.76 | 0.0000 | 4.59, 6.92 |
|  | Presence of glaucoma | No (R) |  |  |  |
|  |  | Yes | 3.04 | 0.0000 | 2.29, 3.78 |
| Normal | Sex | Female (R) |  |  |  |
|  |  | Male | 0.54 | 0.2376 | -0.02 , 1.11 |
|  | Place of residence | Rural (R) |  |  |  |
|  |  | Urban | -0.01 | 0.9920 | -0.55, 0.54 |
|  | Age_cat | 17-39 (R) |  |  |  |
|  |  | 40-64 | -0.66 | 0.0001 | -1.44, 0.11 |
|  |  | > 64 | -0.97 | 0.0001 | -1.8, -0.14 |
|  | Family history | No (R) |  |  |  |
|  |  | Yes | -0.49 | 0.5479 | -1.57, 0.59 |
|  | Type of diabetes | Type 1 (R) |  |  |  |
|  |  | Type 2 | -2.60 | 0.0000 | -3.26, -1.94 |
|  | Hypertension | No (R) |  |  |  |
|  |  | Yes | -0.06 | 0.0995 | -0.89, 0.78 |
|  | Duration of diabetes | 10 years and above (R) |  |  |  |
|  |  | Less than 10 years | 0.97 | 0.0000 | 0.17, 1.77 |
|  | Retinopathy | Non-proliferative (R) |  |  |  |
|  |  | Proliferative | 1.55 | 0.0000 | -0.73, 3.83 |
|  | Maculopathy | No (R) |  |  |  |
|  |  | Yes | 0.44 | 0.0000 | -0.98,1.85 |
|  | Presence of glaucoma | No |  |  |  |
|  |  | Yes | -0.56 | 0.0000 | -1.12, 0.01 |
| Moderate | Base outcome | | | | |
| Severe | Sex | Female (R) |  |  |  |
|  |  | Male | 0.39 | 0.256 | -0.28, 1.07 |
|  | Place of residence | Rural (R) |  |  |  |
|  |  | Urban | 0.03 | 0.9920 | -0.63, 0.68 |
|  | Age_cat | 17-39 (R) |  |  |  |
|  |  | 40-64 | 0.83 | 0.0001 | -0.73, 2.38 |
|  |  | > 64 | 1.4 | 0.0001 | -0.15, 2.95 |
|  | Family history | No (R) |  |  |  |
|  |  | Yes | -0.43 | 0.5479 | -1.74, 0.88 |
|  | Type of diabetes | Type 1 (R) |  |  |  |
|  |  | Type 2 | 1.19 | 0.0000 | 0.09, 2.29 |
|  | Hypertension | No (R) |  |  |  |
|  |  | Yes | -0.19 | 0.0995 | -1.26, 0.86 |
|  | Duration of diabetes | 10 years and above (R) |  |  |  |
|  |  | Less than 10 years | -0.88 | 0.0000 | -1.57, -0.19 |
|  | Retinopathy | Non-proliferative (R) |  |  |  |
|  |  | Proliferative | 2.14 | 0.0000 | -0.15, 4.43 |
|  | Maculopathy | No (R) |  |  |  |
|  |  | Yes | -0.41 | 0.0000 | -2.63, 1.81 |
|  | Presence of glaucoma | No |  |  |  |
|  |  | Yes | 1.54 | 0.0000 | 1.13,1.95 |

R: reference group, (visual impairement= moderate is the base outcome)
