# Supplementary material for: The impact of diabetes on visual acuity in Ethiopia, 2021
Source: PLoS One. 2021 Aug 13;16(8):e0256145. doi: 10.1371/journal.pone.0256145 (PMC8362981; doi:10.1371/journal.pone.0256145)
Supplement: S1 File — (DOCX) [file pone.0256145.s002.docx]

**S1 File. Questionnaire.**

**ጥያቄዎች**

እነዚህ ጥያቄዎች የሚመለከቱት የስኳር በሽተኞች ሆነዉ ከሌላ ጤና ተቋም ወደ ጎንደር ዩኒቨርሲቲ ስፔሻላይዝድ ሆስፒታል ለአይን ህክምና ሪፈር የተላኩትን ሲሆን የተዘረዘሩት መጠይቆች የስኳር በሽታና የአይን እይታ ችግር ተዛማጅነትን የሚመለከቱ ጥያቄዎች ናቸዉ፡፡ በመሆኑም በዚህ ዙሪያ አንዳንድ መረጃዎችን በመሰብሰብ ላይ እገኛለዉ፡፡ ስለሆነም እርስዎ ትክክለኛ መረጃ በመስጠት የበኩለዎን ድርሻ እንዲወጡ ስል በትህትና እየጠየቅኩ መረጃዎቹ ከጥናቱ አላማ ዉጪ እንደማይዉሉ እና ሚስጥራቸዉም በጥብቅ የተጠበቀ እንደሚሆን ቃል እየገባዉ ፍቃደኛ ከሆኑ ወደ ጥያቄዉ እንግባ፡

ሀ) አዎ ፈቃደኛ ነኝ ለ) ፈቃደኛ አይደለዉም

የሚስጥር ቁጥር……… ቀን……………

የስነ ህዝብ ሁኔታ፣የበሽታዉ ባህሪያት እና የምርመራ ዉጤት

1. የተጠያቂዉ እድሜ ……….
2. የተጠያቂዉ ፆታ

ሀ) ወንድ ለ) ሴት

1. የመኖሪያ ቦታ

ሀ) ከተማ ለ) ገጠር

1. የስኳር በሽተኛ ነዎት?

ሀ) አዎ ለ) አይደለዉም

1. በተራ ቁጥር 4 ለተገለፀዉ ጥያቄ መልስዎ አዎ ከሆነ የስኳር አይነትዎ የትኛዉ ነዉ?

ሀ) ታይፕ 1 ለ) ታይፕ 2

1. የስኳር ደረጃዎ የት ላይ ይገኛል?

ሀ) የመጀመሪያ ደረጃ ለ) አድቫንስድ ደረጃ

1. የስኳር በሽታዎ ከሬቲኖፓቲ ወደ ማክሎፓቲ ተለዉጧል?

ሀ) አዎ ለ) አላዉቅም

1. ለምን ያክል ወር/አመት ክትትል አድርገዋል ………….?
2. የደም ግፊት አለብዎ?

ሀ) አዎ ለ) የለብኝም

1. የግላኮማ ችግር አለብዎ ?

ሀ) አዎ ለ) የለብኝም

1. ከቤተሰበዎ መካከል የማየት ችግር ያለበት አለ?

ሀ) አዎ ለ) የለም

1. የዕይታ መጠንዎ የትኛዉ ደረጃ ላይ ነዉ?

ሀ) ኖርማል

ለ) መጠነኛ ችግር አለብኝ

ሐ) የከፋ ችግር አለብኝ

መ) የማየት ችግር አለብኝ

**English version questionnaire**

This questionnaire is prepared for the collection of socio-demography, clinical evaluation, and outcome-related information for diabetic patients who were referred from diabetic clinics to an ophthalmologic evaluation in the University of Gondar comprehensive specialized hospital. The names of the participants were not extracted to ensure the privacy of patient information, and confidentiality was maintained throughout the data collection process and analysis. So, we are requesting your voluntariness to participate and give as information about risk factors for the visual acuity problems. So, are you volunteer to participate? A) Yes B) No

If you are a volunteer precede to the following questions:

Code …………………… Date ……………………….

**Socio-demographic characteristics, clinical and outcome-related variables**

1. Age of the respondents ……………..
2. Sex of the respondents
3. Male B. Female
4. Place of respondents
5. Urban B. Rural
6. Are you diabetic patients?
7. Yes B. No
8. If your answer is yes to question “No. 4”, what type of diabetes that you develop?
9. Type I B. Type II
10. What is the stage of your diabetic retinopathy?
11. Proliferative B. Non-proliferative
12. Is the diabetic retinopathy changed to maculopathy?
13. Yes B. No
14. How many months/ years did you have follow-up ……………..?
15. Did you have hypertension?
16. Yes B. No
17. Did you have glaucoma problems?
18. Yes B. No
19. Did you have any family members who had visual acuity problem?
20. Yes B. No
21. What is your category of visual impairment?
22. Normal C. Severe
23. Moderate D. Blind
