# Supplementary material for: The impact of diabetes on visual acuity in Ethiopia, 2021
Source: PLoS One. 2021 Aug 13;16(8):e0256145. doi: 10.1371/journal.pone.0256145 (PMC8362981; doi:10.1371/journal.pone.0256145)
Supplement: Attachment — Submitted filename: Response to Reviewers.docx [file pone.0256145.s003.docx]

Date 12/7/2021

Mulu Tiruneh

Email: [tirunehmulu1@gmail.com](mailto:tirunehmulu1@gmail.com)

Debre Tabor University

Debre Tabor, Amhara Region, Ethiopia

**Dear editors**, thank you very much for all the comments given regarding our manuscript entitled “The impact of diabetes on visual acuity in Ethiopia, 2021.” which are accepted and included in the revised manuscript. All new changes had highlighted in yellow color in the main manuscript. Here I attached the point-by-point response to the comments. I hope you will get the edited manuscript as per your expectation and I look forwards to hear from you soon.

**Response to Editors**

1. We had reviewed our list of reference, and we tried to correct based on the comments. We did not change the reference lists but, we made complete and corrected the author's name’s

For reference # 1. We add vol.8

For reference # 2. We add the author's name Dandona R

For reference # 3. We add the author's name Bourne R

For reference #t 4. We add the author's name Flaxman SR

For reference # 6. We add the author's name Shaw JE

For reference # 8. We add the author's name Jobert Richie N Nansseu

For reference # 9. We add the author's name Duran S

For reference # 10. We add the author's name Mitchell P

For reference # 11. We add the author's name Andersson D

For reference # 12. We add the author's name Broman A.T

For reference # 13. We add the author's name Gedde SJ

For reference # 14. We add the author's name Willson MR

For reference # 15. We add the author's name Wang Y

For reference # 16. We add the author's name Ferarro J

For reference # 18. We add the author's name Teshome Gobena

For reference # 19. We add the author's name Girsh G. Kamath

For reference # 20. We add the author's name’s, Fekadu Aga and Abdisa Boka

For reference # 21. We add the author's name title MD

For reference # 22. We add the author's name Suto C

For reference # 23. We add the author's name Kadiki OA

For reference # 24. We add the author's name Addisu Wondifraw

2. We had not cited papers that have been retracted. We already used the published articles for our reference lists.

**Response to Reviewer 1**

Based on the reviewer’s comment we have been addressed all comments previously, and they were ensured that all comments had addressed.

**Response to Reviewer 2**

Based on the reviewer’s comment we have been addressed all comments previously, and they were ensured that all comments had addressed.
